# Lineage-selective super enhancers mediate core regulatory circuitry during adipogenic and osteogenic differentiation of human mesenchymal stem cells

**DOI:** 10.1038/s41419-022-05309-3

**Published:** 2022-10-12

**Authors:** Chen Wang, Wen Tian, Shou-Ye Hu, Chen-Xi Di, Chang-Yi He, Qi-Long Cao, Ruo-Han Hao, Shan-Shan Dong, Cong-Cong Liu, Yu Rong, Hua-Feng Kang, Tie-Lin Yang, Zhi Yang, Yan Guo

**Affiliations:** 1 Key Laboratory of Biomedical Information Engineering of Ministry of Education, Biomedical Informatics & Genomics Center, School of Life Science and Technology, Xi’an Jiaotong University, Xi’an, Shaanxi 710049 P. R. China; 2 Honghui Hospital, Xi’an Jiaotong University, Xi’an, Shaanxi 710054 P. R. China; 3 Research and Development Department, Qingdao Haier Biotech Co. Ltd, Qingdao, Shandong 266109 P. R. China; 4 Department of Oncology, The Second Affiliated Hospital of Xi’an Jiaotong University, Xi’an, Shaanxi 710004 P. R. China

**Keywords:** Stem cells, Differentiation, Mesenchymal stem cells

## Abstract

Human mesenchymal stem cells (hMSCs) can be differentiated into osteoblasts and adipocytes. During these processes, super enhancers (SEs) play important roles. Here, we performed comprehensive characterization of the SEs changes associated with adipogenic and osteogenic differentiation of hMSCs, and revealed that SEs changed more dramatically compared with typical enhancers. We identified a set of lineage-selective SEs, whose target genes were enriched with cell type-specific functions. Functional experiments in lineage-selective SEs demonstrated their specific roles in directed differentiation of hMSCs. We also found that some key transcription factors regulated by lineage-selective SEs could form core regulatory circuitry (CRC) to regulate each other’s expression and control the hMSCs fate determination. In addition, we found that GWAS SNPs of osteoporosis and obesity were significantly enriched in osteoblasts-selective SEs or adipocytes-selective SEs, respectively. Taken together, our studies unveiled important roles of lineage-selective SEs in hMSCs differentiation into osteoblasts and adipocytes.

## Introduction

Human mesenchymal stem cells (hMSCs), known as adult multipotent stem cells, have the potential to differentiate into multiple cell lineages, such as osteoblasts and adipocytes [1, 2]. Under normal circumstances, there is a delicate balance between osteogenic and adipogenic differentiation. However, defective differentiation of hMSCs will lead to osteoporosis or obesity-associated diseases [3–5].

Stem cell identity and fate commitment are dominated by lineage-restricted transcription factors (TFs) network and specific epigenetic architectures [6, 7]. Increasing evidence reveal the roles of master TFs in orchestrating the osteogenesis and adipogenesis, such as the promotion role of *ALPL* [8] and *CBFB* [9] for osteogenic differentiation, while *PPARG* [10, 11] and *C/EBPα* [12] for adipogenic differentiation. TFs bind DNA regulatory elements called enhancers, which are key modulators of cell type-specific gene expression programs [13]. Some enhancers group together as clusters, termed super enhancers (SEs) [14], which have been recognized recently with a higher potential to be densely occupied by TFs and their cofactors, and stronger potential to drive gene transcription of key cell identify [13, 15]. During lineage fate commitment, SEs and their dense cluster of TFs binding sites undergo dynamic remodeling, including the disappearance of preexisting SEs and the creation of novel SEs [16]. These newly established SEs are indispensable for the expression of target genes that are required for cell differentiation [17]. Furthermore, SEs could regulate the expression of lineage-determining TFs, which in turn form an interconnected autoregulatory loop termed core regulatory circuitry (CRC) where these TFs not only bind to their own SEs but also to the SEs of other TFs within the loop [18]. Recent studies have revealed that CRC plays pivotal roles in lineage commitment, such as corneal epithelial homeostasis [19]. To date, the mechanisms that SEs and CRC TFs cooperativity in regulating osteogenic and adipocytic differentiation of hMSCs are largely unknown.

Here, using hMSCs as a model, we characterized the SE-mediated regulatory landscape during adipogenesis and osteogenesis. We found that SEs changed more dramatically than typical enhancers (TEs) during hMSCs lineage specification, and identified 251 lineage-selective SEs that regulated pivotal genes expression, especially those key TFs involved in hMSCs commitment. We also demonstrated that lineage-selective SEs had a unique regulatory function in directed differentiation of hMSCs by functional experiments. Furthermore, we found that the lineage-selective SEs mediated the formation of CRC, and meanwhile, the constituent TFs had the potential capacity of binding to most of other lineage-selective SEs and regulated the downstream genes expression. Finally, by integrating the results from genome-wide association studies (GWAS) of osteoporosis and obesity related phenotypes, we detected that 38 GWAS SNPs resided in functional regions of lineage-selective SEs. Machine learning-based in silico prediction uncovered some allele-specific TFs binding events, suggesting the potential pathogenic mechanisms. Overall, this work provides new insights into understanding the determinants of the hMSCs fate determination, and offers novel strategies for the treatment of osteoporosis and obesity.

## Materials and methods

### Data source

H3K27ac, DNase-Seq, RNA-Seq data from hMSCs, adipocytes (hMSC were subjected to adipogentic inducing medium for 7 days), and osteoblasts (hMSC were subjected to osteobalstic inducing medium for 7 days) used in this study were obtained from GEO database under GEO assess number GSE113253.

### H3K27ac ChIP-seq data process

The single-end sequencing reads were aligned to the human hg19 genome using bowtie2 (v 2.3.5) [20] with default parameters. The reads with mapping quality <30 and duplicated reads were filtered. The broad peaks were called by MACS2 (v 2.1.1) [21] with following parameters: --broad --broad-cutoff 0.01.

### DNase-seq data process

The single-end sequencing reads were aligned to the human hg19 genome using bowtie2 (v 2.3.5) [20] with default parameters. The reads with mapping quality <20 and duplicated reads were filtered. The narrow peaks were called by MACS2 (v2.1.1) with following parameters: -q 0.05 --nomodel --extsize 200 --shift −100.

### Super enhancer identification

SEs were identified by H3K27ac ChIP-seq data, the most well-characterized histone mark associated with enhancer activities used to define SEs [22]. All H3K27ac peaks from all samples were merged with bedtools [23] to produce a consistent peak set. We then excluded the promoter peak for later analysis. To do this, we defined the merged peaks that were completely located within the 2 kb upstream and downstream region of transcription start site of protein-coding and lncRNA genes. Gene annotation GTF file was downloaded from gencode portal (gencode v19, https://www.gencodegenes.org/). The remained peaks were regarded as enhancers and used for primary SEs identification of all samples by ROSE2 (https://github.com/linlabbcm/rose2) with default parameters. Next, we redefined the SEs numbers for each sample according to the mean value of all primary SEs number. Finally, the enhancer with a mean rank equal to the mean SEs number (1700) or higher (<1700) in the two replications of the same cell type were considered as SEs for this cell type. The final SEs set is the union of all SEs from the hMSCs, adipocytes or osteoblasts.

### Diff-enhancer analysis

All peaks in consistent peak set with a distance <12.5 kb were stitched together like that be done by ROSE algorithm, then this newly generated stitched peak set was used for all samples as input for DiffBind R package for diff-peak analysis, stitched peaks with FDR ≤ 0.1 (equivalent to the primary *P* = 0.0228) were regarded as diff-enhancer.

### RNA-seq data process

SRA files of RNA-seq were downloaded from SRA database and transformed to fastq format by the command fastq-dump from sra-toolkit. Split-stored RNA-seq data from the same sample were pooled together at the fastq file level. Fastp software was used to filter the sequenced bases with low quality and the remained sequencing reads were aligned to human genome hg19 with hisat2 [24], the FPKM of each gene was calculated by cufflinks [25].

### Lineage-selective SEs identification

Considering the continuum of enhancer signals, especially around the SE/TE threshold, obtaining lineage-selective SEs by simply taking the difference of two SE sets would yield more false-positive results. A possible case is an enhancer region with a mean rank≤1700 in one cell type and with a mean rank>1700 in the other cell type, but with no-significant signal difference between the two cell types. To get lineage-selective SEs with high confidence, we used a combined analysis of enhancer rank and signal difference. For selective-SEs of adipocytes, they should meet following two conditions. Firstly, the mean rank of a specific enhancer in two replicated adipocytes samples should higher than the mean SE number of all samples (≤1700) and its mean rank in two replicated osteoblasts samples should lower than the mean SE number of all samples (>1700). Second, the enhancer should possess significantly higher H3K27ac signal in adipocytes than that in osteoblasts with at least 1.5 fold change. For selective SEs of osteoblasts, the standards above were reversed.

### SEs target genes identification

The SEs target genes were the combination of the target genes of its functional regions identified by the ABC model as described in https://github.com/broadinstitute/ABC-Enhancer-Gene-Prediction. We used ABC model to integrate the histone H3K27ac data and DNase-seq data to identify functional regions of lineage-selective SEs and target genes of them. The collection of genes with ABC scores >0.02 were considered credible target genes.

### Motif enrichment analysis

The functional regions of lineage-selective SEs identified by ABC model were used for motif enrichment by findMotifsGenome.pl subcommand in HOMER with parameter “-size given”. Random genome regions set selected by HOMER was used as background.

### Identification of TF-target pairs mediated by lineage-selective SEs

We used two approaches to identify the TF-target pairs which were mediated by lineage-selective SEs. For each TF-target pair, first, we performed motif scan in the functional regions that targeting the gene with FIMO software, if at least two motifs were found with *P* < 1 × 10^−4^, then this regulatory relationship was considered as truth. Or, the second method was to merge all ChIP-seq peaks of same TF from all available cell lines or tissues in cistrome database, if at least one peak was overlapped with these functional regions, we thought that the gene was regulated by this TF.

### GWAS data

eBMD GWAS summary data was downloaded from GWAS Catalog (https://www.ebi.ac.uk/gwas/home). BMI and BMI adjusted WHR GWAS summary data were downloaded from https://zenodo.org/record/1251813#.YiMlZnpByUl.

### TF-DNA interaction analysis

The GWAS variant (*P* ≤ 5 × 10^−8^) overlapped with functional regions of lineage-selective SEs were used for TF-DNA interaction prediction with QBiC-Preb web server (http://qbic.genome.duke.edu/makeprediction) with default *p*-value cutoff and all available TFs provided by QBiC-Preb. Only the results with status change is “unbound>bound” or “bound>unbound” were retained. According to the GWAS effect, TF-target expression correlation, and bound status change, the results with opposite directions were filtered (Fig. 6D). Two TFs from the QBiC-Preb results, SREBF1 and RBPJ, which have pre-computed DeltaSVM models using ENCODE TF ChIP-seq data (https://www.beerlab.org/deltasvm_models/), were reanalyzed with score_snp_seq.py script from https://www.beerlab.org/deltasvm_models/.

### In vitro cell culture and differentiation

The frozen primary human umbilical cord derived hMSCs were obtained from Shaanxi Stem Cell Engineering Co., Ltd from 1 donor who have signed the informed consent for this study. HMSCs were maintained according to manufacturer’s instructions using mesenchymal stem cell growth medium (Yinfeng, Xi’an, Shaanxi, China). HMSCs of the 3rd-5th generation were used for cell experiments.

To induce osteogenic differentiation, different groups of hMSCs were cultured in the basic medium with 10% FBS (Invitrogen, Waltham, MA, USA), 1% penicillin/streptomycin, 10 mM glycerol-2-phosphate (Sigma, St. Louis, MO, USA), 50 μM L-ascorbic acid (Sigma), and 100 nM dexamethasone (Sigma) for 7 days. For adipogenic differentiation induction, when the hMSCs achieved 100% confluence, different groups of hMSCs were subjected to adipogenesis differentiation kit (Cyagen, Taicang, Suzhou, China) according to the manufacturer’s instructions for 7 days.

HEK 293 T cells were from Chinese Academy of Sciences and were cultured in DMEM supplemented with 10% FBS (Invitrogen, Carlsbad, CA, USA) and 1% penicillin/streptomycin at 37°C in a humidified atmosphere of 5% CO_2_.

### ALP staining and Nile red staining

After 7 days of osteogenic induction, cells were washed three times with PBS and fixed in 4% paraformaldehyde for 30 min. Alkaline phosphatase staining was performed according to the Alkaline Phosphatase Assay Kit (Beyotime, Haimen, Jiangsu, China). After 7 days of adipogenesis induction, cells were washed three times with PBS and fixed in 4 % paraformaldehyde for 60 min. Nile red staining was performed according to the manufacturer’s instructions (Thermo Fisher Scientific, Waltham, MA, USA).

### Plasmid construction and viral infection

To efficiently delete the functional regions residing in lineage-selective SEs, we performed CRISPR/Cas9 genome editing experiments as described previously [26]. Briefly, guide RNAs were designed by the Zhang Lab (https://zlab.bio/guide-design-resources), then inserted into lentiCRISPR v2 plasmid (Addgene, plasmid no. 52961) as described in our previous report [27]. These recombinant plasmids were used for lentivirus package. SgRNAs primers were listed in Supplementary Table S7.

To repress genes expression, we applied CRISPR/dCas9 mediated technology. In brief, we first designed sgRNAs at CRISPick website (https://portals.broadinstitute.org/gppx/crispick/public), these sgRNAs were annealed and ligated into the BsaI enzyme site of pUC19- hU6-sgRNA backbone vector and then cut by Kpn1 and Spe1. Finally, the cutted fragment was cloned into ourself constructed KRAB-expressing vector.

The transfer plasmid was co-transfected with packaging plasmid psPAX2 (Addgene, plasmid no. 12260) and envelope plasmid pCMV-VSV-G (Addgene, plasmid no. 8454) into HEK 293 T cells for 48 h to produce lentivirus. The supernatant medium containing target lentivirus was collected and filtrated by 0.22 μM aperture PES membranes (Millipore, Darmstadt, Hesse, Germany). Then hMSCs were infected with lentivirus suspension mixed with 8 ug/ml polybrene (Santa Cruz Biotechnology, Dallas, TX, USA). Two days after infection, 1 ug/ml puromycin was added to select sgRNA/ Cas9-positive cells for further research.

### RNA isolation, reverse transcription, and quantitative real-time PCR

Total RNA was extracted using TRIzol (Invitrogen, Waltham, MA, USA) reagent according to manufacturer’s protocol. 1 μg of total RNA was then reverse transcribed to cDNA using SuperScripts II First-Strand cDNA Synthesis Kit (Invitrogen, Waltham, MA). Quantitative real-time PCR (qRT-PCR) was conducted with the QuantiTect SYBR^®^ Green PCR Kit (Qiagen, Dusseldorf, NRW, Germany) on Bio-Rad instrument. The 2^−ΔΔCT^ method was used for data analysis. The qPCR primers are listed in Supplementary Table S7.

### Chromatin immunoprecipitation assay

Chromatin immunoprecipitation was performed using an SimpleChIP^®^ Enzymatic Chromatin IP Kit (Magnetic Beads) (Cell Signaling Technology, Danvers, MA, USA). Pre-cleared chromatin was immunoprecipitated with mice monoclonal antibodies against ETS1 (sc-55581, Santa Cruz Biotechnology, Dallas, TX, USA) or MEF2A (sc-17785, Santa Cruz Biotechnology, Dallas, TX, USA), normal rabbit IgG or antibody against acetyl Histone H3 (included in the kit). The total cell chromatin extract was used as input. Protein-DNA crosslinks were precipitated by using ChIP-Grade Protein G Magnetic Beads. And DNA is purified using DNA purification spin columns (included in the kit). Following quantification was undertaken by quantitative PCR analysis with primers listed in Supplementary Table S7.

### Statistical analysis

Statistical analysis was performed using GraphPad Prism software (GraphPad Software, Inc., La Jolla, CA, USA) and R language. The data are displayed as means ± standard deviation. Experiments were repeated independently at least three times. Data were analyzed for significance of difference by Student’s *t*-test. A value of *P* < 0.05 was considered significant.

## Results

### SEs changed more dramatically than TEs during hMSCs adipogenic and osteogenic differentiation

**Fig. 1:**
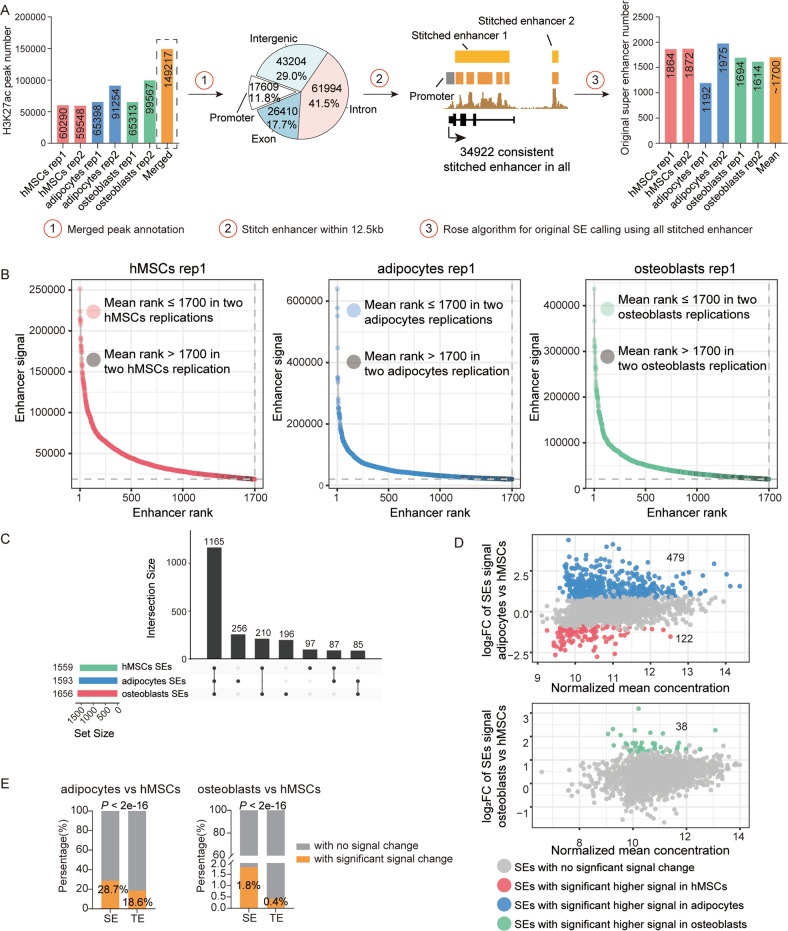
Enhancer change in the process of hMSCs differentiation. **A** Pipeline for SEs identification. The H3K27ac peaks from each sample were merged to generate a consistent peak set comprise 149217 peaks. After excluding of 17609 promoter regions, remained enhancers within a genome distance of 12.5 kb were stitched together and resulted in a total of 34922 stitched enhancers. Next, ROSE algorithm was applied to each sample to determine a primary SE number. The mean value of primary number from all samples was used to redefine the SE number for each sample. The enhancers with a mean rank of 1700 or higher (<1700) in each cell type were considered as SEs for this cell type. **B** Scatter plots display SE signal and rank of hMSCs, adipocytes and osteoblasts from the first replication of differentiation experiment. The intersection points of dashed line corresponding to the cut-off point of SEs and TEs, in *X*-axis, it corresponding to 1700. Colored points indicate SEs with a mean rank of 1700 or higher (<1700) in two replications, while gray points indicate SEs with a mean rank lower (>1700) in two replications. **C** Upset plot display the SE number for each cell type and the overlap among three or between two cell types. **D** Scatter plots display SE signal alteration between differentiated cells and hMSCs: up panel showed the signal alteration of SEs between adipocytes and hMSCs, the lower panel showed the signal alteration of SEs between osteoblasts and hMSCs. Blue dots indicate a significant higher (FDR ≤ 0.1, equivalent to the original *P* ≤ 0.0228) signal in adipocytes cells, green dots indicate a significant higher signal in osteoblasts, red dots indicate a significant higher signal in hMSCs. **E** Bar plots display the percentage of enhancers with significant signal change after differentiation into adipogenic and osteogenic lineage.

To investigate whether SEs play roles in the lineage commitment process of hMSCs osteogenic and adipogenic differentiation, we first detected the alteration of SEs signals in undifferentiated hMSCs, osteoblasts and adipocytes, separately. SEs were identified by H3K27ac ChIP-Seq data. We called H3K27ac peaks for each sample and later conducted peak merging, promoter excluding, enhancer stitching and original SEs calling (Fig. 1A). We redefined the SEs numbers for each sample as the mean of all original SEs numbers from ROSE algorithm, here, was 1700. Then, the enhancers with mean rank of 1700 or higher (<1700) in two replications of the same cell type were considered as SEs for the cell type (Fig. 1B, S1A). From this, we got 1656 SEs for hMSCs, 1593 SEs for adipocytes and 1559 SEs for osteoblasts (Fig. 1C), respectively. Most SEs were shared among all three cell types (Fig. 1C). We next explored the alteration of H3K27ac signals at enhancers between differentiated hMSCs and un-differentiated hMSCs. Similar to TEs (Fig. S1B), SEs showed significant signal change after differentiation induction (Fig. 1D), despite the osteoblasts did not change as much as adipocytes, which has been proven to be a feature of hMSCs differentiation [28]. Notably, we observed more dramatic change of SEs than that of TEs after differentiation (Fig. 1E), suggesting the driver function of enhancers in the process of hMSCs differentiation, especially more varied SEs.

### Lineage-selective SEs were associated with hMSCs lineage commitment

**Fig. 2:**
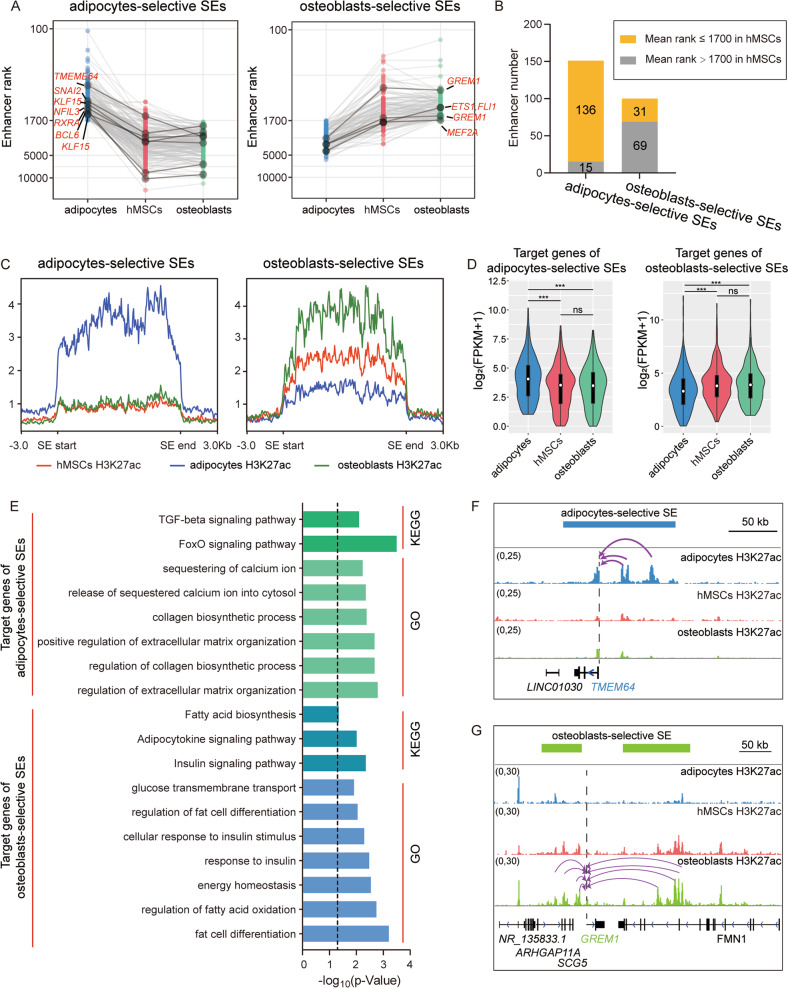
Identification of lineage-selective SEs and functional enrichment of their target. **A** Scatter plots show the mean rank of adipocytes- (left panel) and osteoblasts-selective SEs (right panel) in three cell types, the same genome region in three cell types were connected by lines, some examples were marked with dark gray, the target genes of these examples were also showed. **B** Bar plot shows the counts of lineage-selective SEs which with a signal rank ≤1700 in hMSCs. **C** The H3K27ac signal profile of lineage-selective SEs in three cell types. All SE regions were extended to a consistent length and the surrounding 3 kb regions were also plotted in figures. **D** Violin plots show expression of adipocytes-selective SEs (left panel) and osteoblasts-selective SEs (right panel) in three cell types and the statistical difference among them; ***P < 0.001, ns: not significant. **E** Bar plot of significant enriched (*P* ≤ 0.05) GO and KEGG terms of genes regulated by adipocytes- or osteoblasts-selective SEs. The dashed line corresponding to the significance threshold. **F**, **G** Genome browser plots show examples of adipocytes-selective SE (**F**) and osteoblasts-selective SE (**G**) with their targets. Dashed line indicates the position of TSS of target genes. The arc with arrow links the functional region within SEs and TSS of target gene, which was predicted by ABC model.

Next, to investigate whether there were lineage-selective SEs involved in hMSCs lineage commitment, we first generated a lineage-selective SEs set with high confidence by exploring the difference of SEs between osteoblasts and adipocytes with a combined analysis of SEs signal rank and strength (Method). We identified 100 and 151 lineage-selective SEs in osteoblasts and adipocytes lineages, respectively (Fig. 2A, Supplementary Table S1-2). Comparing the rank and signal of these lineage-selective SEs in hMSCs, our results indicated that the adipocytes-selective SEs showed a tendency of de novo establishment, while the osteoblasts-selective SEs were more likely to be inherited from the already existing SEs in hMSCs just with a signal increase in osteoblasts lineage and a signal decrease in adipocytes lineage (Fig. 2A–C). Specifically, 90.1% (136/151) of adipocytes-selective SEs possessed a higher mean rank (≤1700) in hMSCs (Fig. 2B), and exhibited very weak H3K27ac signals in hMSCs (Fig. 2C). But for osteoblasts-selective SEs, 69.0% (69/100) possessed a lower mean rank (>1700) in hMSCs (Fig. 2B), and the H3K27ac signals of these regions were already high in hMSCs, but showed an opposite tendency between adipocytes and osteoblasts lineages (Fig. 2C).

To further reveal the role of these lineage-selective SEs in lineage commitment, we predicted the functional regions of these lineage-selective SEs and their target genes with Activity-by-Contact (ABC) model [29], which integrated H3K27ac ChIP-seq and chromatin accessibility data. In total, 442 and 318 target genes were identified for 123 adipocytes- and 82 osteoblasts-selective SEs, respectively (Supplementary Table S1–2). As expected, target genes of adipocytes-selective SEs showed significantly higher expression in adipocytes compared to that in hMSCs and osteoblasts, while target genes of osteoblasts-selective SEs showed significantly higher expression in osteoblasts than that in adipocytes, but not in hMSCs (Fig. 2D). Functional enrichment analysis showed that target genes of adipocytes-selective SEs enriched in multiple biological progresses/signaling pathways linked to adipocyte characteristics, such as fatty acid biosynthesis, regulation of fat cell differentiation, and response to insulin [30] (Fig. 2E). Similarly, target genes of osteoblasts-selective SEs were mainly involved in osteoblasts formation progress, such as TGF-β signaling pathway [31, 32], collagen biosynthetic process, and positive regulation of extracellular matrix organization (Fig. 2E). As an example, here we showed *TMEM64*, which was regulated by an adipocytes-selective SE (Fig. 2A, F), could accelerate the adipogenesis and restrain the osteogenesis by modulating Wnt/β-catenin signaling [33]. Another example suggested the regulatory role of two osteoblasts-selective SEs to *GREM1* expression (Figs. 2A, 2G). The expression of *Grem1* marked a population of osteochondroreticular (OCR) stem cells that could differentiate into osteoblasts but not adipocytes [34], and the inactivation of *Grem1* in mice resulted in severe skeletal development defects [35]. Together, these results indicated that lineage-selective SEs exerted their lineage commitment effects by regulating the key genes crucial for the hMSCs fate choice.

### TFs associated with lineage-selective SEs played critical roles in hMSCs fate decision

**Fig. 3:**
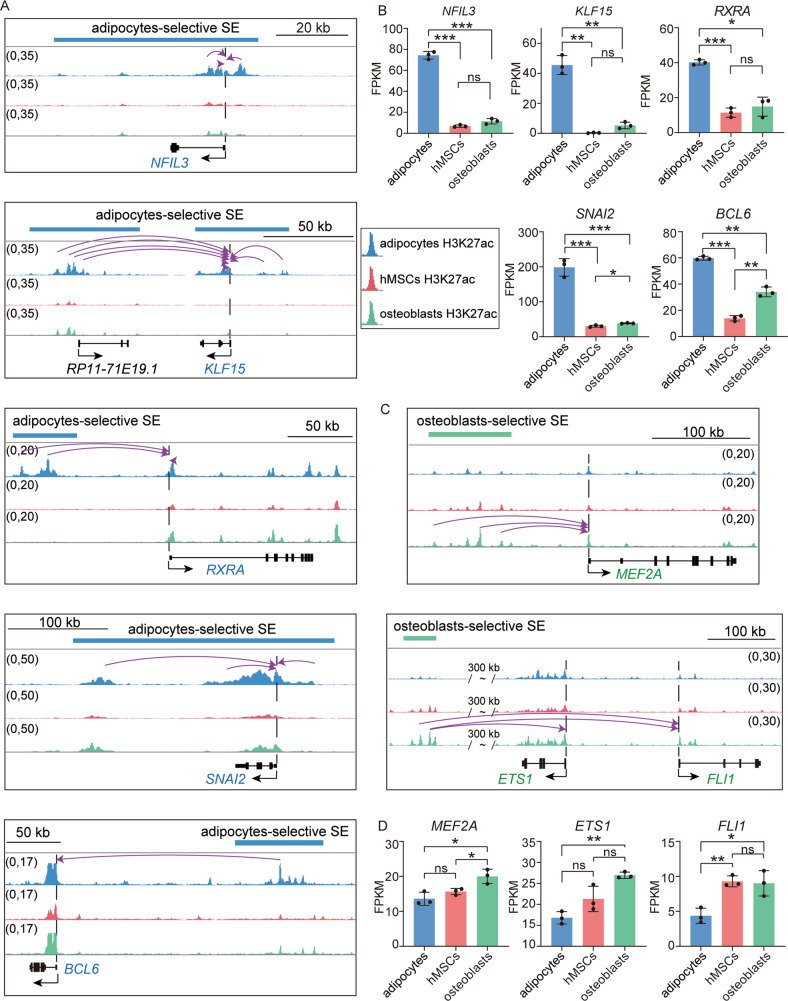
TFs targeted by lineage-selective SEs and the expression. **A** Genome browser plots display adipocytes-selective SEs and their target TFs. Dashed line indicates the TSS of gene. The arc with arrow links the TSS and the functional region within SEs that predicted to target it. **B** Bar plots display mean values ± S.D. of FPKM of TFs regulated by adipocytes-selective SEs from *n* = 3 replications of three cell types and the statistical difference; *P < 0.05, **P < 0.01, ***P < 0.001, ns: not significant. **C** Genome browser plots display osteoblasts-selective SEs and their target TFs. Dashed line indicates the TSS of gene. The arc with arrow links the TSS and the functional region with in SEs that predicted to target it. **D** Bar plots display mean values ± S.D. of FPKM of TFs regulated by osteoblasts-selective SEs from *n* = 3 replications of three cell types and the statistical difference; *P < 0.05, **P < 0.01, ***P < 0.001, ns: not significant.

Increasing evidence supports SEs as key elements defining cell identity by regulating target TFs [14, 36, 37]. But whether and how SE-TF pairs control the process of hMSCs differentiation have not been fully answered. Therefore, we focused on the target TFs which were regulated by lineage-selective SEs and showed significantly differential expressions between adipocytes and osteoblasts. For adipocytes-selective SEs, we identified five target TFs, including *NFIL3*, *KLF15*, *RXRA*, *SNAI2*, and *BCL6* (Fig. 3A, B), which were significantly up-regulated in adipocytes rather than osteoblasts or hMSCs (Fig. 3B). For osteoblasts-selective SEs, we identified three target TFs, including *MEF2A*, *FLI1*, and *ETS1* (Fig. 3C), with significantly high expression in osteoblasts than adipocytes (Fig. 3D). All these TFs have been reported to be involved in imbalance of hMSCs lineages commitment or the resulting phenotypes/diseases [28, 38, 39]. For example, *NFIL3* regulated by an adipocytes-selective SE has been reported as a key pro-adipogenic TF to promote adipogenesis and related to glucocorticoid-regulated adipogenesis [40, 41]. *MEF2A* targeted by an osteoblasts-selective SE, which was in consistent with previous study [39], was accompanied with expression of osteogenic markers during osteogenesis [28].

### Genome knockout of lineage-selective SEs revealed their indispensable and specific roles in hMSCs differentiation

**Fig. 4:**
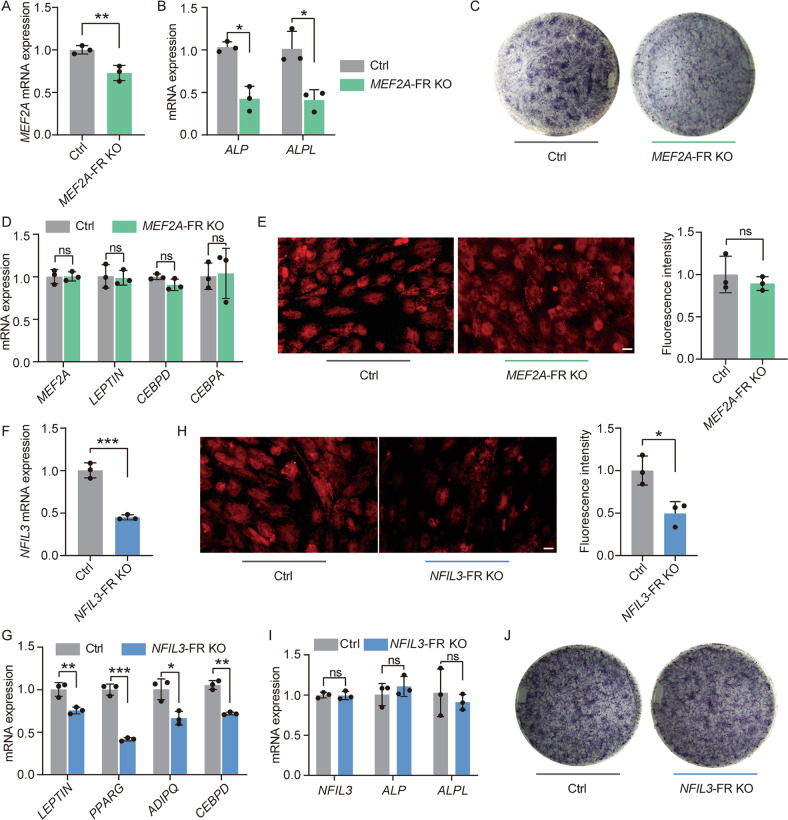
Functional validation of lineage-selective SEs. **A**, **B** RT-qPCR analysis of gene expression normalized to *GAPDH* expression level. The results were presented as means ± S.D. *P < 0.05, **P < 0.01. **C** Representative images show ALP staining in hMSCs with or without knockout the FR of osteoblasts-selective SE that targeting *MEF2A*. **D** RT-qPCR analysis of gene expression normalized to *ACTIN* expression level. ns: not significant. **E** Representative images show nile red staining in hMSCs after 7 days of adipocytic differentiation with or without knockout the FR of *MEF2A* osteoblasts-selective SE. Images were captured and fluorescence intensity was quantitated using Image J software. The percentage fluorescence intensity relative to control is shown and expressed as mean ± S.D. (*n* = 3). Scare bar: 100 μm. ns: not significant. **F**, **G** RT-qPCR analysis of gene expression normalized to ACTIN expression level. *P < 0.05, **P < 0.01, ***P < 0.001. **H** Representative images showed nile red staining in hMSCs after 7 days of adipocytic differentiation with or without the FR of *NFIL3* ADI-lineage SE. Images were captured and fluorescence intensity was quantitated using Image J software. The percentage fluorescence intensity relative to control is shown and expressed as mean ± S.D. (*n* = 3). Scare bar: 100 μm. *P < 0.05. **I** RT-qPCR analysis of gene expression normalized to *GAPDH* expression level. ns: not significant. **J** Representative images show ALP staining in hMSCs with or without knockout the FR of osteoblasts-selective SE that targeting *NFIL3*.

To further validate the regulatory relationship of lineage-selective SE-TF pairs and the effect exerted by that on the lineage commitment, we used CRISPR-Cas9 genome editing system to knock out the core functional region (FR) which had the highest H3K27ac signal of two lineage-selective SEs targeting *MEF2A* and *NFIL3* in hMSCs, separately (Fig. 3A, C). Then, the knock-out cells (*MEF2A*-FR-KO and *NFIL3*-FR-KO) and control cells (hMSCs) were subjected to both osteoblastic and adipogenic differentiation synchronously. After treatment with osteoblastic medium for 7 days, the expression of *MEF2A* was significantly decreased in *MEF2A*-FR-KO cells compared with control group (Fig. 4A), resulting in significantly inhibition of osteoblastic differentiation, which were supported by the decreased *ALP* and *ALPL* expression and lower ALP stain signal in knock-out cells compared with control cells (Fig. 4B, C). In order to explore whether osteoblasts-selective SEs affected adipogenic differentiation, we treated *MEF2A*-FR-KO cells in adipogenic cocktail for 7 days. The qRT-PCR result showed that the expression of *MEF2A* and the adipogenetic markers (*CEBPA, LEPTIN, PPARG* and *CEBPD*) were unchanged compared with control group (Fig. 4D). Nile red staining showed no difference in lipid accumulation signals between knock-out and control cells (Fig. 4E). These results served as an example to support the indispensable role of osteoblasts-selective SEs in osteoblastic differentiation, but not in adipogenic differentiation.

Similarly, after adipogenetic induction, the *NFIL3*-FR-KO cells showed significantly lower expression of *NFIL3* compared with control (Fig. 4F), accompanied by the downregulation of adipogenic makers (*LEPTIN, PPARG, ADIPQ* and *CEBPD*) (Fig. 4G). Nile red staining results revealed that adipogenesis progress was significantly disrupted in *NFIL3-*FR-KO cells (Fig. 4H). While treated with osteogenic medium for 7 days, *NFIL3*-FR-KO cells and negative control cells showed no difference in expression of osteogenesis makers and ALP staining (Fig. 4I, J). These observations indicated the key role of adipocytes-selective SE in adipogenic differentiation, but not in osteoblastic differentiation.

### Lineage-selective SEs mediated the formation of core regulatory circuitry

**Fig. 5:**
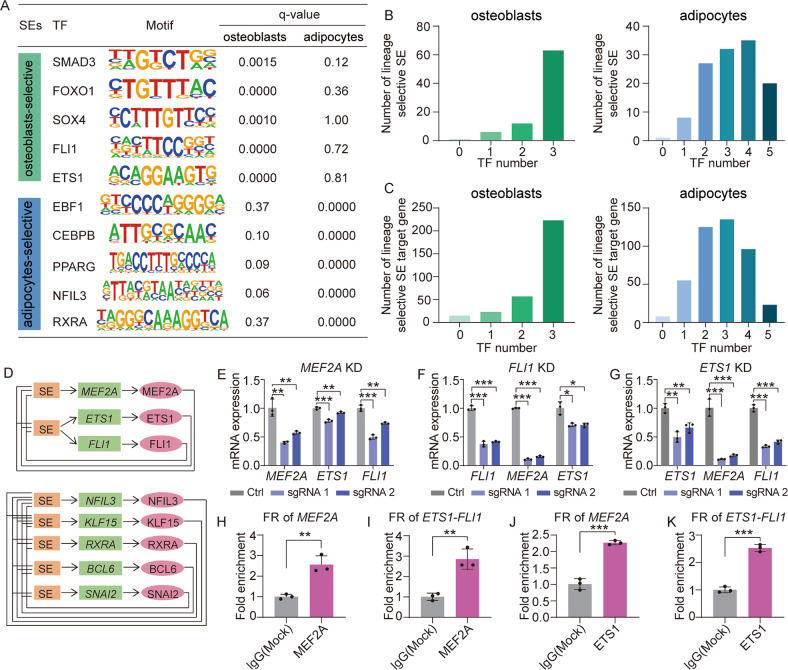
Lineage-selective SEs mediated the formation of CRC. **A** Table depicts TFs binding motifs that specifically enriched in functional regions of adipocytes- or osteoblasts-selective SEs and corresponding q-values. **B** Bar plots display the number of lineage-selective SEs bound by a specific number of TFs. **C** Bar plots display the number of target genes of lineage-selective SEs regulated by a specific number of TFs. **D** In silico predicted CRC constituted by TFs regulated by lineage-selective SEs. **E–G** RT-qPCR analysis of gene expression between control and target gene knockdown cells. *GAPDH* was used as loading control. *P < 0.05, **P < 0.01, ***P < 0.001. **H**, **I** Bar plot of ChIP-qPCR result displays the *MEF2A* binding efficiency at osteoblasts-selective SEs of *MEF2A* and *ETS1/FLI1*. **P < 0.01. **J**, **K** Bar plot of ChIP-qPCR result displays the *ETS1* binding efficiency at osteoblasts-selective SEs of *ETS1/FLI1* and *MEF2A*. ***P < 0.001.

To explore the upstream supervisor of lineage-selective SEs, we performed the TF motif enrichment analysis in functional regions of the two lineage-selective SEs sets with HOMER [42]. As illustrated in Fig. 5A, the key TFs that have been widely proved to be involved in osteogenesis (*SMAD3* [43], *FOXO1* [44], *SOX4* [45]) and adipogenesis (*PPARG* [46], *EBF1* [47], *CEBPB* [48]) were specifically enriched in osteoblasts-selective and adipocytes-selective SEs sets, respectively, suggesting the vital roles of master TFs in the establishment of SEs. Notably, among these enriched TFs, four TFs (*FLI1* and *ETS1* for osteoblasts, *NFIL3* and *RXRA* for adipocytes) were also regulated by lineage-selective SEs (Fig. 3A, C), suggesting the governing roles of these TFs in promoting hMSCs differentiation by binding to lineage-selective SEs and driving the expression of their targets, but not just as downstream genes as other non-TF target genes of lineage-selective SEs.

Therefore, we then focused on the TFs regulated by lineage-selective SEs and explored their binding capacity on the lineage-selective SEs (Method). We observed that almost all the lineage-selective SEs were potentially bound by at least one lineage-selective SEs-regulated TF (Fig. 5B), as a consequence, almost all the lineage-selective SEs targeted genes were under control of these TFs (Fig. 5C). Especially, we found that the lineage-selective SEs mediated the formation of a regulatory network among their targeted TFs. For osteogenic lineage, a fully connected CRC was constructed (Fig. 5D, up panel). In which, 3 TFs including *MEF2A*, *ETS1* and *FIL1* were osteoblasts-selective SE-regulated and these 3 TFs themselves bind to the osteoblasts-selective SEs of one another. To directly interrogate their interconnected transcriptional regulation, each TF in the CRC of osteoblasts was silenced using CRISPR/dCas9-KRAB inhibition (CRISPRi) system. We found that knockdown of any single TF significantly decreased the expression of the other two members (Fig. 5E–G). Our ChIP-qPCR results further showed the enrichment of MEF2A at the lineage-selective SE region of itself and that of *ETS1* and *FLI1* (Fig. 5H, I). Similarly, Fig. 5J, K showed the enrichment of ETS1 at the lineage-selective SE region of itself and that of *MEF2A* and *FLI1*. Together, these data identified and validated an interconnected CRC consisting of three lineage-selective SEs-regulated core TFs in osteoblasts. In addition, we also established a CRC consisting of *NFIL3*, *KLF15*, *RXRA*, *SNAI2*, and *BCL6* in adipocytes (Fig. 5D, lower panel). Most of the TFs in this CRC were co-expressed with each other using population-based gene expression data from adipose tissues [49, 50] (Fig. S2), indicating the reliability of CRC regulatory network identified in adipocytes lineage.

### GWAS variants in functional region of lineage-selective SEs

**Fig. 6:**
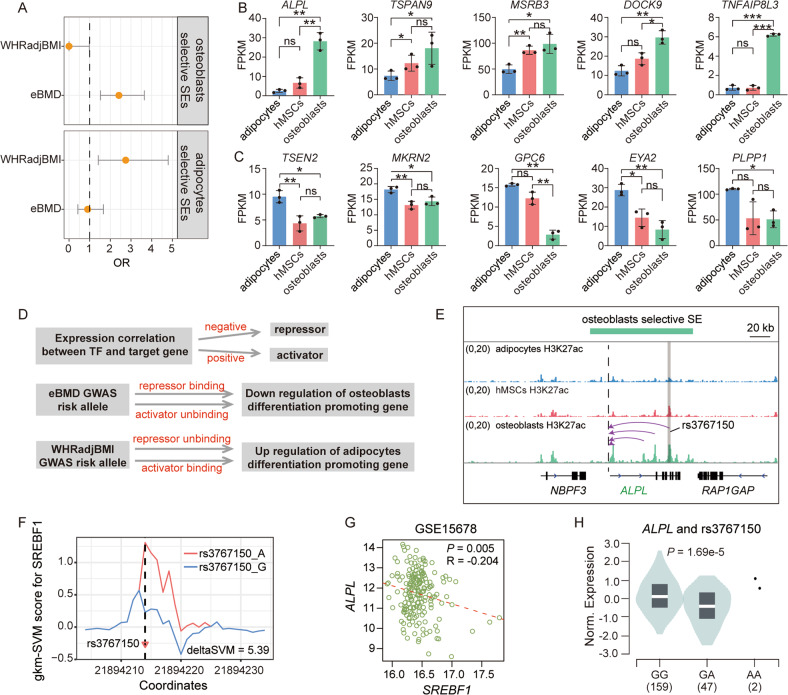
GWAS variants located in lineage-selective SEs. **A** Enrichment results of eBMD and WHRadjBMI GWAS variants in the functional regions of two lineage-selective SEs sets. Odds ratios are presented with their 95% CI. Enrichments were calculated using Fisher’s exact test (two-sided). **B**, **C** Bar plots display mean values ± S.D. of FPKM and the statistical difference; *P < 0.05, **P < 0.01, ***P < 0.001, ns: not significant. **D** Schematic diagram displays the mechanism hypothesis through which the GWAS variants function. **E** Genome browser plot displays a lineage-selective SE in osteoblasts that regulate *ALPL*. Dashed line indicates the TSS of *ALPL*. The arc with arrow links the TSS and the functional region within SE that predicted to target it. An eBMD associated GWAS variant, rs3767150 reside in one of the functional regions of this SE. **F** The 11-mer gkm-SVM scores across rs3767150 for SREBF1. **G** The expression correlation of *SREBF1* and *ALPL* in osteoblast (GSE15678). **H** The violin plot displays the expression of *ALPL* in samples with different genotype of rs3767150 from GTEx liver tissue.

Increasing studies, including ours, revealed that a plethora of variants related to osteoporosis and obesity resided in enhancers [51–53], which might lead to the imbalance adipo-osteogenic differentiation of hMSCs [54]. Therefore, we hypothesized that the GWAS variants located in functional regions of hMSCs lineage-selective SEs were more likely to be causal variants. We then focused on the variants that were located within the functional regions of lineage-selective SEs and associated with osteoblasts or adipocytes related phenotypes. We found that the GWAS variants (*P* ≤ 5 × 10^−8^) associated with estimated bone mineral density (eBMD) [55] were significantly enriched in the functional regions of osteoblasts-selective SEs (OR = 2.41, *P* = 2.30 × 10^−4^), while the GWAS variants of waist-to-hip ratio adjusted for body mass index (WHRadjBMI) [56] were significantly enriched in the functional regions of adipocytes-selective SEs (OR = 2.74, *P* = 1.96 × 10^−3^) (Fig. 6A). Specifically, 23 eBMD-associated SNPs within the functional regions of osteoblasts-selective SEs were linked to 15 genes (including 12 protein-coding genes and three long non-coding genes) (Supplementary Table S3). Among which, five genes showed significantly higher expression in osteoblast than that in adipocytes (Fig. 6B), and some genes have been proven to be important for osteogenic commitment, such as *ALPL*, which was a common-accepted marker of osteogenic differentiation [8]. The potential causal role of these SNPs were supported by expression quantitative trait loci (eQTL) data from Genotype-Tissue Expression (GTEx) project [50] (Supplementary Table S3). On the other hand, there were 13 WHRadjBMI associated GWAS variants located in five adipocytes-selective SEs and targeting 6 protein coding genes and a long non-coding gene (Supplementary Table S4). In addition, there were also two BMI associated variants located in a adipocytes-selective SE and targeting *C10orf11*. eQTL data also corroborated most of the regulatory relationships between these GWAS variants and their target genes (Supplementary Table S4). Despite the lack of functional evidence for these target genes in the process of adipogenic differentiation, almost all of them showed significantly higher expressions in adipocytes compared to that in hMSCs and osteoblasts (Fig. 6C), strongly indicating their critical roles in adipogenic lineage commitment.

Disruption of transcription factor binding sites is a common way in which GWAS variants in enhancer regions exert their effect. Therefore, we used QBiC-Pred [57], a prediction method based on regression models of DNA-binding specificity trained on high-throughput in vitro data and could give statistical report for the effect of TF-DNA binding status change, to predict the impact of GWAS variants on the transcription factors-DNA interactions. By fitting the regulatory hypothesis of GWAS risk allele (Fig. 6D), we identified probable TF bond status change events between alleles of 9 GWAS variants (4 eBMD GWAS variants and 5 WHRadjBMI GWAS variants), involving five target genes (one osteoblasts-selective SE regulated gene *ALPL* and 4 adipocytes-selective SEs regulated genes including *C10orf11*, *TSEN2*, *MKRN2* and *EYA2*) (Supplementary Table S5–6). These findings may uncover the mechanism by which GWAS variants function. To verify the results of QBiC-Pred, we used another effective gkm-SVM based method to predict the effect of TF-DNA binding with pre-computed 11-mer sequences weight [58, 59]. For SREBF1 and RBPJ, which were predicted as effector TFs in QBiC-Preb results, also had pre-computed 11-mer sequences weight for gkm-SVM model. For eBMD associated rs3767150, located in an osteoblasts-selective SE and targeting *ALPL* (Fig. 6E), gkm-SVM score confirmed the QBiC-Pred result that the allele G of rs3767150 could disrupt the binding of SREBF1 on this region (Fig. 6F). While SREBF1 was a potential repressor of *ALPL* (Fig. 6G), this is consistent with that the allele G was a promoting genotype for the expression of *ALPL* in GTEx data (Fig. 6H) and with a positive contribution to the eBMD (Supplementary Table S5). Taken together, our data provide an entry point for further pathogenesis studies of these GWAS variants.

## Discussion

The potential of hMSCs to differentiate into osteoblasts and adipocytes is partly driven by enhancer regulation, and previous studies have made remarkable achievements in this research area [16, 28, 41]. For example, Rauch et al. found that osteoblasts differentiation mainly depends on the activation of enhancers that have been established in hMSCs, conversely, adipogenesis involves chromatin remodeling and *de novo* establishment of enhancers [28]. However, they didn’t explore the differences of enhancers between osteoblasts and adipocytes in detail. Here, we divided enhancers into TEs and SEs, and demonstrated that SEs changed more acutely than TEs during hMSCs differentiation. Further, we identified a set of osteoblasts-selective and adipocytes-selective SEs, respectively. Similar with Rauch et al. study in general enhancers [28], our results revealed that osteoblasts-selective SEs were pre-existed in hMSCs whereas adipocytes-selective SEs were nascent upon adipocytic induction. The functions of their target genes were highly lineage-specific and differed in biological functions, which may explain how SEs regulate precise gene expression patterns during hMSCs fate determinant. For example, an osteoblasts-selective SEs target gene, *ALPL* (Supplementary Table S1), has been widely verified in osteogenesis [8]. Besides the genes we mentioned in this study, we also provided an extensive catalog of genes regulated by lineage-selective SEs (Supplementary Table S1-2), which is a useful resource for future functional characterization on hMSCs fate choice.

Multiple master TFs and SEs could form CRC to promote communication between TFs in a cooperative manner [14], which play important roles in determining tissue specificity, such as human embryonic stem cells [60] and limbal stem/progenitor cells [19]. Here, we established osteogenic and adipogenic CRCs, respectively. The osteogenic CRC includes 3 TFs, *MEF2A*, *ETS1* and *FLI1*, whose mRNA levels were significantly correlated with each other and could promote each other’s expression by interacting with their lineage-selective SEs. Especially, we functionally validated that knockdown of *MEF2A* could repress osteoblasts formation which is in line with a previous study, that reduction of *MEF2A* expression inhibits osteogenic differentiation but promotes adipogenic differentiation [28]. Moreover, *ETS1* could activate osteopontin expression, which is responsible for directing adipogenesis and osteogenesis from hMSCs [61, 62]. Another member *FLI1*, its reduction also blocks osteogenesis as a previous study mentioned [28]. On the other hand, we established an adipocytic CRC, consisting of *NFIL3*, *KLF15*, *RXRA*, *SNAI2*, and *BCL6*. Nevertheless, whether this CRC regulates adipogenesis is worthy of further investigation.

GWASs have identified thousands of loci associated with human complex diseases/traits. However, >90% of SNPs are in the non-coding region [14], it is a great challenge to parse their functions. Previous studies have suggested that SNPs associated with specific diseases are particularly detected in the SEs of disease-relevant cell/tissue types [14, 63]. In this study, we found that GWAS variants from osteoporosis or obesity related phenotypes specifically enriched in the adipocytes- or osteoblasts-selective SEs, respectively, suggesting the potential important role of these SEs GWAS variants. As a further exploration of mechanism of these SE resided GWAS variants, we predicted the effect on the TF binding of them by two machine learning based methods. By this, our study provides a direction for the functional interpretation of these GWAS variants, and the future functional experiments are worth to be performed.

In conclusion, by establishing a map of lineage-selective SEs in hMSCs differentiation into osteoblasts and adipocytes, our study is expected to improve our understanding for the mechanism of hMSCs differentiation balance and provide an attractive insight into the development of a therapeutic strategy for the osteoporosis and obesity.

## Supplementary information

supplemental figures

Table S1

Table S2

Table S3

Table S4

Table S5

Table S6

Table S7

checklist- CDDIS-22-2447R

## Data Availability

The data that support the findings of this study is freely available from the GEO database under code GSE113253.
