# Supplementary material for: Lineage-selective super enhancers mediate core regulatory circuitry during adipogenic and osteogenic differentiation of human mesenchymal stem cells
Source: Cell Death Dis. 2022 Oct 12;13(10):866. doi: 10.1038/s41419-022-05309-3 (PMC9556616; doi:10.1038/s41419-022-05309-3)
Supplement: Supplementary file 1 — supplemental figures [file 41419_2022_5309_MOESM1_ESM.pdf]

Fig S1

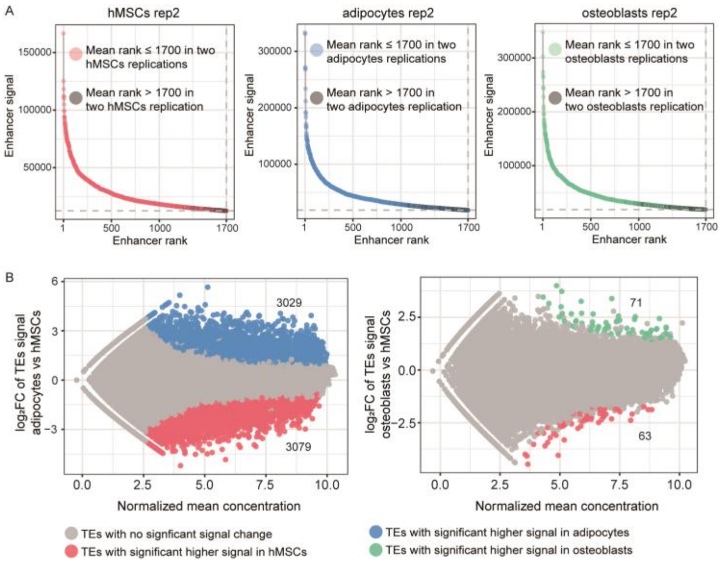

Fig. S1 Rank and signal of super enhancer in each sample

A Scatter plots display SE signal and rank in hMSCs, adipocytes and osteoblasts cells from the second replication of the differentiation experiment. The intersection points of dashed line corresponding to the cut-off point of SEs and TEs, in X axis, it corresponding to 1700. Colored points indicate SEs with a mean rank of 1700 or higher (<1700) in two replications, while grey points indicate SEs with a mean rank lower (>1700) in two replications. **B** Scatter plots display TE signal alteration between differentiated cells and hMSCs: up panel showed the signal alteration of TEs between adipocytes and hMSCs, the lower panel showed the signal alteration of TEs between osteoblasts and hMSCs. Blue dots indicate a significant higher (FDR ≤ 0.1, equivalent to the original $P \leq 0.0228$) signal in adipocytes cells, green dots indicate a significant higher signal in osteoblasts, red dots indicate a significant higher signal in hMSCs.

Fig S2

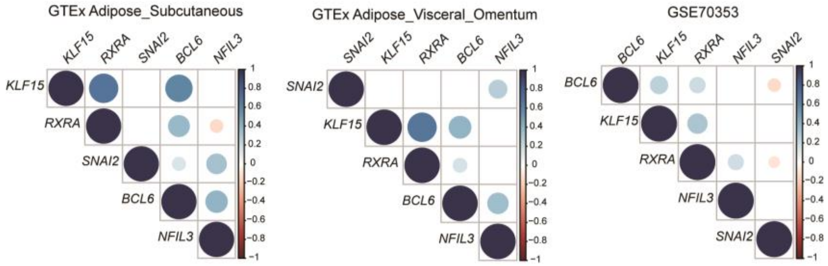

Fig. S2 Expression correlation of TFs regulated by adipocytes-selective SEs in adipose tissue

Correlation plot display the expression correlation among TFs regulated by adipocytes-selective SEs in adipose tissues.
